# Development of an HSV-1 neutralization test with a glycoprotein D specific antibody for measurement of neutralizing antibody titer in human sera

**DOI:** 10.1186/s12985-016-0508-4

**Published:** 2016-03-18

**Authors:** Yong Luo, Dan Xiong, Huan-Huan Li, Sheng-Ping Qiu, Chao-Long Lin, Qin Chen, Cheng-Hao Huang, Quan Yuan, Jun Zhang, Ning-Shao Xia

**Affiliations:** State Key Laboratory of Molecular Vaccinology and Molecular Diagnostics, National Institute of Diagnostics and Vaccine Development in Infectious Diseases, School of Public Health, Xiamen University, Xiamen, 361102 China; School of Life Sciences, Xiamen University, Xiamen, 361102 China

**Keywords:** Herpes Simplex virus type I, Neutralizing antibody, ELISPOT, Cohort study

## Abstract

**Background:**

Investigating the neutralizing antibody (NAb) titer against HSV-1 is essential for monitoring the immune protection against HSV-1 in susceptible populations, which would facilitate the development of vaccines against herpes infection and improvement of HSV-1 based oncolytic virotherapy.

**Results:**

In this study, we have developed a neutralization test based on the enzyme-linked immunospot assay (ELISPOT-NT) to determine the neutralizing antibody titer against HSV-1 in human serum samples. This optimized assay employed a monoclonal antibody specifically recognizing glycoprotein D to detect the HSV-1 infected cells. With this test, the neutralizing antibody titer against HSV-1 could be determined within one day by automated interpretation of the counts of cell spots. We observed good correlation in the results obtained from ELISPOT-NT and plaque reduction neutralization test (PRNT) by testing 22 human serum samples representing different titers. Moreover, 269 human serum samples collected from a wide range of age groups were tested, the average neutralizing antibody titer (log_2_NT_50_) was 8.3 ± 2.8 and the prevalence of NAbs was 83.6 % in this cohort, it also revealed that the average neutralizing antibody titer in different groups increased with the age, and no significant difference in neutralizing antibody titers was observed between males and females.

**Conclusions:**

These results prove that this novel assay would serve as an accurate and simple assay for the assessment of the neutralizing antibody titers against HSV-1 in large cohorts.

## Background

Herpes simplex virus type 1 (HSV-1) is the causative pathogen of orolabial herpes, one of the most prevalent oral transmitted diseases worldwide. More than 60 % of people worldwide were infected with HSV-1 during their lifetime [1, 2]. HSV-1 infection may become chronic, which may lead to oral vesicular lesions at the sites of primary infection, and is recognized as a significant cause of morbidity and mortality in the newborn and immunocompromised individuals [3, 4]. Moreover, HSV-1 also accounts for a substantial proportion of genital herpes infections [5]. HSV-1 viruses cannot be eradicated, which can establish latency for lifelong in sensory ganglia following initial acquisition, thus the majority of people infected with HSV-1 stay in a state of “asymptomatic”.

Generation of humoral immunity and cellular immunity was indicated to play a crucial role in controlling herpes virus shedding [6, 7]. Neutralizing antibodies (NAbs) to viral glycoproteins were elicited in response to HSV-1 infection [8], which may provide some certain protection against HSV-1 acquisition or reduce the severity of HSV-1 related diseases [9, 10]. Surveillance of seroprevalence against HSV-1 in susceptible populations is essential for monitoring the seroconversion rates in countries where HSV-1 is endemic to evaluate the effectiveness of vaccination and anti-viral therapy against herpes infection, especially to HSV-2 infection. Moreover,HSV-1 based oncolytic virus, which usually contain attenuated modifications and expresses multiple therapeutic genes, had been proved to be good therapeutic gene therapy vectors for treating central nervous disease and multiple cancers [11, 12]. NAbs against HSV-1 are one of the barriers oncolytic viruses encounter during viral replication in vivo. However, it is largely unknown whether the circulating NAbs in cancer patients will restrict the efficacy of HSV-1 based virotherapy, especially when the viruses are administrated through intravenous delivery [13]. Thus, it’s intriguing for us to know the average NAb titers in general population, the relationship between HSV-1 serostatus and NAb titers, and which amount of NAbs will affect the efficacy of HSV-2 vaccine, as well as HSV-1 based oncolytic virotherapy.

Conventional assays previously employed to quantitatively determine the NAb titers against herpes virus include the cytopathic effect-based neutralization test (CPE-NT) and the plaque reduction neutralization test (PRNT) [14, 15], which are widely accepted and considered the laboratory gold standard test. However, these assays are arduous and time consuming, requiring long time of infections and plenty of manual counting jobs. Thus, these tests may not be feasible to screen large cohorts from clinical trials or epidemiology studies. Therefore, a high-throughput assay is needed for rapid and precise evaluation of the NAb titers against HSV-1.

Then ELISPOT is a powerful tool for detection and quantification of single secreting cell in a 96 well plate. The ELISPOT with high sensitivity has been successfully applied to detect low frequency cells secreting cytokines, antigens and antibodies, which is widely used in immunological studies and vaccine development [16–18]. Recently, the ELISPOT assay has been adapted to measure the NAb titers against various viruses, such as HIV, enterovirus and dengue virus [19–21]. In this study, we established a high-throughput, quantitative and reliable ELISPOT-based neutralization test (ELISPOT-NT) to rapidly measure the neutralizing antibody titer against HSV-1 in human serum samples. Employing this test, we depicted the prevalence of NAbs among a cohort with wide range of ages.

## Results

### Characterization of the MAbs in this study

**Fig. 1:**
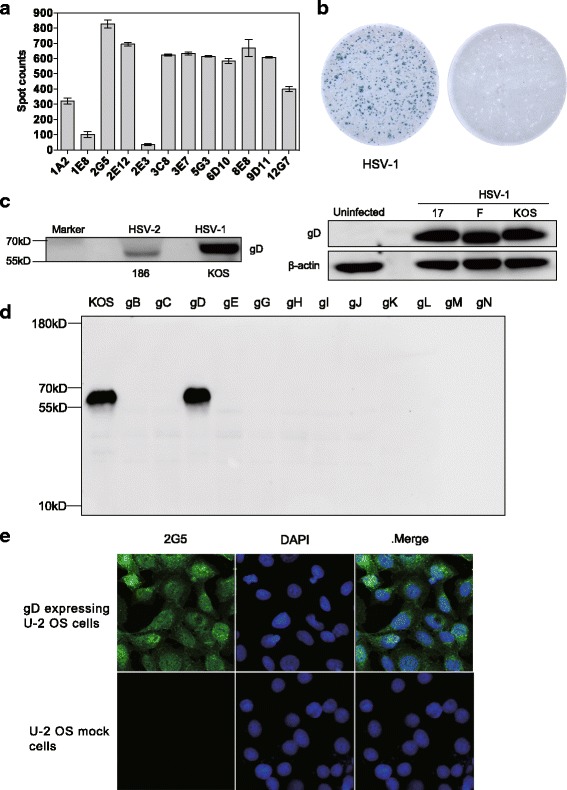
Specificity of MAbs in detecting the HSV-1 infected cells. **a** 12 MAbs were selected from a panel of 60 antibodies developed in our laboratory for specifically recognizing the HSV-1 infected cells. The Cell based ELISPOT assay was used in the detection. **b** Schematic image of the 2G5 antibody in detecting the HSV-1 (KOS strain) infected cells and mock-infected cells, the shown blue spots indicate the viral infection. **c** The reactivity of 2G5 against other HSV viruses. U-2 OS cells were infected with HSV-2 (Strain 186), HSV-1 (Strain 16, Strain F, Strain KOS), cells were harvested 48 h after transfection and total protein was extracted and analyzed by Western Blot. **d** The reactivity of 2G5 against HSV-1 glycoproteins. U-2 OS cells were transfected with 12 glycoprotein constructs (plv-gB ~ gN). Cells were harvested 48 h after transfection and total protein was extracted and analyzed by Western Blot. KOS infected cell lysate was taken as a control. **e** Immunofluorescence analysis of the specificity of 2G5 MAb. After plv-gD transfection and puromycin selection, gD expressing U-2 OS cells were established and stained with 2G5 MAb. U-2 OS mock cells transfected with empty lentivirus vector and selected with puromycin were analyzed as a control

To develop MAbs with high reactivity toward HSV-1 virus, we selected 12 MAbs from more than 60 MAbs that were developed in our laboratory, which can specifically bind to HSV-1 infected cells by ELISPOT assay. Among these 12 MAbs, 2G5 MAb showed with highest sensitivity against HSV-1 compared with other antibodies when tested at the same antibody concentration (Fig. 1a), so 2G5 MAb was selected as the detection antibody. It was observed that 2G5 specifically reacted with HSV-1 infected cells rather than control cells, and legible spots could be formed for subsequent analysis (Fig. 1b). It was found that 2G5 harbored good reactivity with other HSV-1 strains, such as strain F and strain 17, and cross-reacted weakly with HSV-2 (Fig. 1c). 12 viral glycoproteins (gB ~ gN) were employed to determine the target protein of 2G5 MAb, it revealed that 2G5 MAb specifically recognized denature or naïve form of glycoprotein D (gD) without any background (Fig. 1d and e).

### Influence of infection parameters on the detection of cells infected with HSV-1

**Fig. 2:**
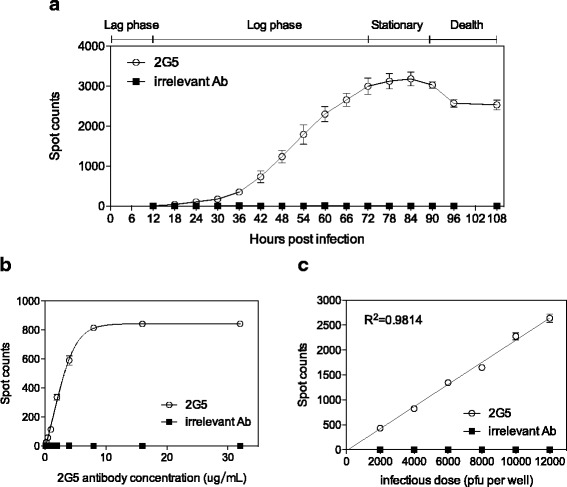
Optimized detection for HSV-1. **a** Infection kinetics of HSV-1. The number of spots during HSV-1 infection at a MOI of 0.005 was monitored for 108 h. The average numbers of spots were calculated from triplicate experiments, and the error bars show the standard deviations. **b** Spot detection with different dilutions of 2G5 MAb. The monolayer U-2 OS cells were infected with KOS at a MOI of 1 × 10^4^ PFU, then detected by serially diluted 2G5 detection antibody. **c** Relationship between the infectious dose and the number of spots. The number of spots was obtained from triplicate infection under a series of infectious doses ranged from 2000 PFU to 12000 PFU. An irrelevant antibody was used as a negative control

**Fig. 3:**
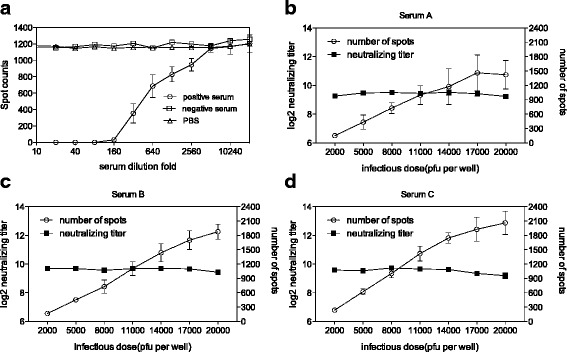
Feasibility of ELISPOT-NT. **a** The assessment of two independent serum samples by ELISPOT-NT. Number of spots obtained from serial dilutions of one positive serum and one negative serum was shown. For the ELISPOT-NT, the incubation time was set to 14 h and the infectious dose was set to 1 × 10^4^ PFU. **b** Influence of the infectious dose on the accuracy of ELISPOT-NT. The neutralizing antibody titers (NT_50_) of three independent serum samples (designated as A, B and C) and the number of spots was measured under different infectious doses. All of the NT_50_ values were log_2_-transformed. Results represent mean ± SD from three independent experiments

The ELISPOT neutralizing test was based on the use of 2G5 to detect the cells infected with HSV-1, thus the optimization of experimental parameters, such as infection time, antibody concentration and infectious dose, was a crucial step in the establishment of 2G5 based ELISPOT assay. First, we depicted the infection kinetics of HSV-1 in U-2 OS cells in order to determine the optimized infection time, when we can precisely quantify individual spots. U-2 OS cells were infected with KOS at a MOI of 0.005, then the number of spots was counted every 6 h for a total of 108 h. As shown in Fig. 2a, the growth curve of HSV-1 was divided into four phases: Lag phase (0–12 h), Log phase (12–72 h), Stationary phase (72–90 h) and Death phase (90–108 h). It was speculated that the virus might go through an entire life cycle within 12 h post infection. The expression of gD protein usually occurred in the mid/late stage of viral infection, so the infection time for detection was set as 14 h. Next, 2G5 MAb (1.6 mg/mL) was firstly started with a 50-fold dilution, then 2-fold serial dilutions of 2G5 MAb were tested by their reactivity with HSV-1 infected cells, the maximum number of spots were not affected when antibody concentration ranged from 8 μg/mL to 32 μg/mL were used in the detection (Fig. 2b). Thus the working concentration of 2G5 MAb was set as 8 μg/mL. Moreover, we investigated the role of infectious dose on the detection of HSV-1 infection. Interestingly, it was observed that the spot count was indeed lower than the number of input viruses. Infection with high MOI and infectious deficiency may account for this phenomenon. However, a good correlation between infectious doses and spot counts (*R*^2^ = 0.9814) was observed in Fig. 3c, the linear range of the infectious doses was 2000 PFU to 12000 PFU. Due to the good correlation between the spot counts and the input viruses, the 2G5 based ELISPOT assay might be further developed to measure the neutralizing antibody titers of serum samples.

### Feasibility of the ELISPOT-NT

Based on the previous findings, we next evaluated the feasibility of ELISPOT based neutralizing test (ELISPOT-NT) using 2G5 antibody on determining the neutralizing antibody titers of human serum samples. To verify this, we first selected a NAb-positive serum sample, a NAb-negative serum sample and a negative control (PBS) for testing. As shown in Fig. 3a, it was found that the spot counts decreased along with the increasing folds of dilution in the NAb-positive serum, while there was no any inhibition effect in the NAb-negative serum.

Moreover, it was necessary to investigate whether the neutralizing antibody titers of human serum samples can be reliably measured by use of ELISPOT-NT under different experimental variations, such as infectious dose. Thus, three independent serum samples were measured with the ELISPOT-NT under different infectious doses. As shown in Fig. 3b, c and d, the results obtained from the ELISPOT-NT were repeatable and consistent, the infectious doses did not influence the neutralizing antibody titers (NT_50_) of three independent serum samples. Due to the limit of serum usage, determining the neutralizing antibody titer become difficult for serum samples with NT_50_ below 5. Thus, if the NT_50_ of a serum sample (both IgG and IgM negative) was below 5, it would be defined as negative (unpublished data).

### Correlation between PRNT and ELISPOT-NT

**Fig. 4:**
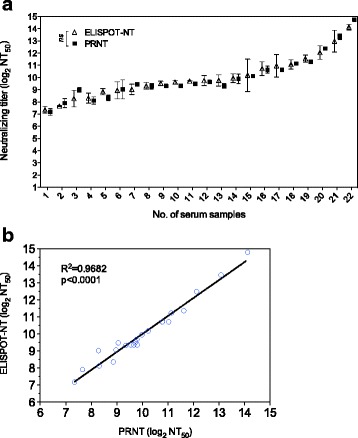
Comparison of the PRNT and ELISPOT-NT. **a** The neutralizing antibody titers of 22 human serum samples, representing different titers, were assayed by PRNT and ELISPOT-NT independently. The average NT_50_ of each sample by these two tests was log2-transformed and shown in side by side. The testing serum samples were arranged from low titer to high titer. **b** The average NT_50_ value of each serum sample that was assayed by the ELISPOT-NT was plotted against the average NT_50_ value of corresponding serum sample that was assayed by the PRNT. The concordance between ELISOT-NT and PRNT was shown by linear regression analysis. Results represent mean ± SD from three independent experiments

The PRNT is the standard method for the measurement of NAb titers against HSV-1. Therefore, we evaluated the concordance between the PRNT and ELISPOT-NT. 22 serum samples representing different neutralizing antibody titers were selected and tested in three independent experiments using both PRNT and ELISPOT-NT assay. As shown in Fig. 4a, the NAb titers of 22 serum samples measured by ELISPOT-NT were almost equal to the those obtained from PRNT. There was a good correlation between these two assays by linear regression analysis (*R*^2^ = 0.9682) (Fig. 4b). Since the NT_50_ values tested by the ELISPOT-NT assay were consistent with those tested by classical PRNT assay, it indicated that the ELISPOT-NT had good repeatability and reliability. In conclusion, the ELISPOT-NT can be used to measure the NAb titers against HSV-1 in human serum samples.

### Characteristics of study participants

Using the ELISPOT-NT, we screened a cohort consisting of hundreds of serum samples to test its applicability, and dissected the prevalence of NAbs against HSV-1 in general population. This study enrolled 269 healthy individuals from Xiamen city (Table 1). Participants were aged 1–59 (mean: 30.0 ± 17.1) years and included 126 males and 143 females. Participants were divided into five age groups. The study samples were representative of the general population from Xiamen, a coastal city of China.

**Table 1:** Baseline characteristics of a cohort from general population in Xiamen

Group	*n* (%)	HSV-1 seroprevalence (%)	HSV-1 NAb prevalence (%)	Average NAb titers (log_2_NT_50_)
Age (years)				
≤10	41 (15.2 %)	29.3 % (17.6 %-44.5 %)*	31.7 % (19.6 %-47.0 %)*	4.4 ± 3.1
11-20	47 (17.5 %)	74.5 % (60.5 %-84.8 %)*	74.5 % (60.5 %-84.8 %)*	7.5 ± 3.1
21-30	41 (15.2 %)	92.7 % (80.6 %-97.5 %)	95.1 % (83.9 %-98.7 %)	9.2 ± 1.6
31-40	49 (18.2 %)	91.8 % (80.8 %-96.8 %)	97.9 % (89.3 %-99.6 %)	9.1 ± 1.4
41-50	48 (17.8 %)	91.7 % (80.5 %-96.87 %)	97.9 % (89.1 %-99.6 %)	9.5 ± 1.7
>50	43 (16.0)	97.7 % (87.9 %-99.6 %)	100 % (91.8 %-100 %)	9.8 ± 1.6
Gender				
Male	126 (46.8 %)	83.3 % (75.9 %-88.8 %)	84.9 % (77.7 %-90.1 %)	8.2 ± 2.8
Female	143 (53.2 %)	76.9 % (69.4 %-83.1 %)	82.5 % (75.5 %-87.9 %)	8.4 ± 2.9
Total	269	79.9 % (74.7 %-84.3 %)	83.6 % (78.8 %-87.6 %)	8.3 ± 2.8

*p < 0.05; 95 % confidence interval shown in brackets

### The prevalence of NAbs against HSV-1 in general population

**Fig. 5:**
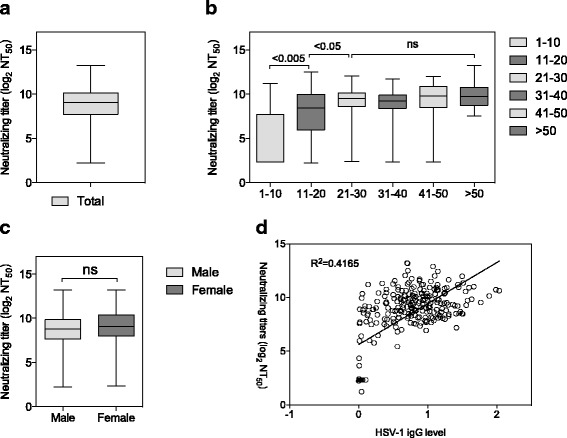
Application of ELISPOT-NT on assessing the neutralizing antibody titers against HSV-1 in a cohort. **a** The overall NAbs titers of this cohort were shown as log_2_ (NT_50_). **b** The average NAb titers among different age groups. This cohort was divided into five age groups as shown. The differences between bars 1 and 2 (*p* < 0.005) and between bars 2 and 3 (*p* < 0.05) were statistically significant by Mann–Whitney test. In contrast, no statistically significant difference was found among bars 3, 4, 5 and 6 (*p* > 0.05). **c** The average NAb titers between males and females. The NAb titers of each sex or age group were shown in box and whiskers, which shows the minimum, first quartile, median, third quartile and maximum titer levels. **d** Relationship between the anti-HSV IgG level and the NAb titers of 269 serum samples. The anti-HSV-1 IgG values of this cohort were tested by commercial anti-HSV-1 IgG ELISA kit, the NAb titers were assayed by ELISPOT-NT and were shown as log_2_ (NT_50_). 10 sera from this cohort were confirmed with HSV-1 IgG negative but NAbs Positive

The seroprevalence rate of HSV-1 infection was 79.9 % in this cohort (215/269), which was slightly higher in males. Due to the high seroprevalence, the prevalence of NAbs against HSV-1 was calculated and found to be 83.6 % (225/269), with average NAb titers (log_2_NT_50_) of 8.3 ± 2.8 (Fig. 5a). Comparing the neutralizing antibody titers against HSV-1 among different age groups, the average NAb titers increased with the ages, a significant difference in NAb titers was observed among groups with age younger than 30, but there was no significant difference among groups with age greater than 20 (Fig. 5b). No significant difference in NAb titers was observed between males and females (Fig. 5c). The anti-HSV-1 IgG level of individual was not fairly consistent with the level of NAb titers in this cohort (*R*^2^ = 0.4165). Interestingly, all individuals tested with HSV-1 IgG positive were presenting with HSV-1 NAb positive, but 10 individuals tested with HSV-1 IgG negative by ELISA were presenting with NAb positive, possibly due to the missed detection of HSV-1 ELISA assay (Fig. 5d).

## Discussion

Although PRNT has been accepted worldwide as the gold standard for measuring NAb titers against viruses, it is also known that some deficiencies of PRNT, such as time-consuming and labor-intensive, restricts the usage of this method in high throughput screening. Therefore, some novel assays for titration of NAbs had been developed, such as neutralization assays based on a reporter gene, flow cytometry and ELISPOT [22, 23]. Recently, ELISPOT-NT have showed some advantages in assessing the NAb titer with higher sensitivity and better concordance with PRNT than other assays, which relied on the antibodies to recognize the infected cells that express specific viral proteins [16, 20, 21]. Usually, the viral proteins selected need to be abundantly expressed during the initial infection and be accessible by the antibodies. The secondary antibodies coupled to horseradish peroxidase can further improve the sensitivity of the assay by enzyme amplification effect. To our knowledge, a feasible ELISPOT-NT for detecting NAbs against herpes virus had not been established before. Therefore, we screened a panel of MAbs against HSV-1 to test if they can detect the HSV-1 infected cells with high specificity and sensitivity. We selected an antibody, named 2G5 and specifically recognized the glycoprotein D, which could be used for detection of HSV-1 infection. Based on this antibody, we established a quantitative and reliable ELISPOT assay for determining the NAb titers. Factors that may impact the accuracy of this assay has been systematically evaluated, it turns out that this assay is reproducible and consistent within a broad linear range of infectious doses. The theory that the neutralizing antibody titers are not impacted by the infectious doses is simply the reflection of “the percentage law”, which states that if the antibody concentration is way above the virus concentration, a given concentration of antibody neutralizes the same relative proportion of virus infectivity irrespective of the quantity of virus present [24, 25].

Taking the results of PRNT as the reference, ELISPOT-NT gave similar results in testing the neutralizing antibodies among all representing 22 sera. A good correlation (*R*^2^ = 0.9682; *P* < 0.0001) was observed between PRNT and ELISPOT-NT. Compared with the PRNT, the ELISPOT-NT is an efficient neutralization test with several advantages: it is rapid and the whole procedure can be finished within 24 h, from the time cells are seeded into plates until the data is analyzed; it is more flexible, the infectious dose within a relatively wide range has little influence on the results; it is precise, the titration of NAbs is repeatable and consistent with the result obtained from PRNT; it is high throughput, thousands of samples can be tested in a single run. Recently, Blevins et al. reported a high throughput assay to quantify the NAb titer against herpes simplex viruses in human serum samples, this assay employed a ELVIS cell line, which was stably transfected with the LacZ gene under the control of the HSV ICP6 promoter [26]. Under the circumstance of HSV infection, the LacZ protein was expressed and could be detected by addition of CPRG substrate. The NAb titer could be consistently quantified using this assay, but the correlation data between this assay and classical PRNT assay was not reported. ELISPT-NT requires less operation time than ELVIS cell-based assay (24 h vs. 46 h). However, both these two assays are not fully automated, require 2 ~ 3 h’s plate manipulation.

Protection against HSV-1 infection correlates well with the antibody levels, whereas the exact role for NAbs in controlling HSV-1 infection and reflecting T-cell responses remains elusive [27, 28]. ELISPOT-NT shows potential as a powerful method for screening neutralization activities of large numbers of serum samples. From a cohort of general population with different ages in Xiamen, we observed a significant increasing trend in the average NAb titers along with the age, suggesting that the general population were suffered from lifelong challenge by HSV-1 virus. For the first time, we found that HSV-1 serologic status was not fully related to the NAb titers, 10 individuals tested with HSV-1 IgG negative by ELISA were presenting with NAb positive, which implied that the different clinical significances for anti-HSV-1 IgG level and the neutralizing antibody titers. A reasonable explanation is there are several types of neutralizing antibodies against more than five HSV-1 proteins, but the current HSV-1 IgG ELISA kits may only target one or two major ones. Thus, further learning the level of NAbs response to infection in general populations is important for understanding how these protective Abs may affect the HSV-1 recurrence, including their significance in the development of vaccines against herpes virus.

Although HSV-1 harbors a high similarity with HSV-2, the biologic characteristics of them are obviously different. The development of a vaccine to prevent HSV-2 infection had been failed due to inconsistently effective results in people with different serologic status of herpes virus acquired from the clinical trails [29]. Interestingly, in women who were negative for antibodies to both HSV-1 and HSV-2, HSV-2 gD-2 vaccine seemed to protect a small proportion of them from HSV-1 infection rather than HSV-2 infection [3]. However, the factor contributes to this type-specific protection remains largely unknown. A reasonable explanation is there are some differences in the antibody activity against HSV-1 and HSV-2. To prove this point, the NAb titers of vaccine-induced antibodies against HSV-1 should be determined. This setback in the development of HSV-2 vaccine provides some important information for future design of new generation vaccines, which can elicit higher titers of NAbs than those induced by natural infection, or induce stronger T cell response [8].

The HSV-1 based oncolytic virus, Talimogene laherparepvec, had been confirmed to be therapeutically potent in a phase 3 trial of treating metastatic melanoma and got the approval of FDA into market in 2015 [30, 31]. Additionally, more than 5 HSV-1 based oncolytic viruses had also been proved to be safe and effective in the different clinical trials against multiple cancers [32–35]. NAbs against HSV-1 are one of the barriers viruses encounter during viral replication in vivo [36]. However, it is largely unknown whether the circulating NAbs in cancer patients will restrict the efficacy of HSV-1 based oncolytic virotherapy, especially when the viruses are administrated through intravenous delivery [13]. A survey we conducted in the general population by testing the NAb titers against HSV-1 revealed that the average NAb titers were 8.3 ± 2.8, and the prevalence of NAbs against HSV-1 increased with age, with an older population (above 50 years old) having the highest NAb titers (the average neutralizing antibody titers was 9.8 ± 1.6). As we know, older adults have a higher risk for cancer, it’s intriguing for us to know whether the NAbs will reduce the efficacy of oncolytic virus mediated cancer remission in patients with higher NAb titers, especially among those with elder ages. Further studies are still needed to design for supporting the importance of NAbs against HSV-1 so as to broaden the application of this ELISPOT-NT.

## Conclusions

We established an HSV-1 neutralization test based on the enzyme-linked immunospot assay (ELISPOT-NT) to determine the neutralizing antibody titer against HSV-1 in human serum samples. In this test, we employed a monoclonal antibody specifically recognizing glycoprotein D to detect the cells that were infected with HSV-1. Using this high-throughput test, the neutralizing antibody titers against HSV-1 from human sera can be detected precisely within 24 h. The stability and consistency of this assay was systematically evaluated in parallel with a conventional neutralization test, a good correlation in determining the neutralizing antibody titers was observed. Through a cohort study, in the general population consisting of 269 healthy individuals in Xiamen City of China, we observed significant differences in neutralizing antibody titers across the different ages by using this test. We also demonstrated that the anti-HSV-1 IgG level was not closely associated with neutralizing antibody titers. The ELISPOT-NT test could serve as an accurate and simple assay for large cohort studies in assessing the role of the neutralizing antibody titers against HSV-1 on the development of vaccines against herpes infection and HSV-1 based oncolytic virotherapy.

## Methods

### Cells and Viruses

Human U-2 OS cells and HEK 293 T cells were purchased from American Type Culture Collection (ATCC) and maintained in Dulbecco’s modified Eagle’s medium (DMEM) containing 10 % fetal bovine serum (FBS), 100 U/mL penicillin, and 100 μg/mL streptomycin. U-2OS cells are widely recognized as a common cell model for HSV-1 titration and functional study of HSV-1 infection [37–39]. Besides, U2OS cells are a good cell option for spot detection due to their characteristics of big size and round shape. Herpes simplex virus 1, KOS strain, was purchased from ATCC. Viruses were propagated and titrated in U-2 OS cells, and kept in storage at −80 °C.

### Plasmids and transfection

Lentivirus vector was purchased from Addgene. The HSV-1 glycoprotein genes (gB ~ gN) were amplified from KOS genome and subcloned into lentivirus vector. Lentivirus constructs with glycoprotein-expressing genes (plv-gB ~ gN) were co-transfected respectively in 293 T cells along with helper vectors using Lipofectamine 2000 according to the manufacturer’s instructions (Invitrogen). For viral transduction, the U-2 OS cells were transduced with the medium containing lentivirus harvested 48 h after transfection, in the presence of 6 μg/mL polybrene. Stable clones were selected under the pressure of puromycin.

### Human serum samples

Serum samples from 269 healthy individuals with various ages were collected at random times throughout 2014 from the Center for the Disease Control and Prevention (CDC) of Xiamen. All study participants resided in the city of Xiamen.

### ELISA

Total antibodies against HSV-1 in this cohort was determined by HSV-1 IgG ELISA kit (Wantai Bio). Tests were performed as described in the manufacturer’s instruction manuals. In brief, 100 μl of a 5-fold diluted serum sample was added to the microplate well and incubated at 37 °C for 1 h. After stringent washing, and then 100 μl of HRP-conjugated secondary antibody was added and incubated 37 °C for 0.5 h. The wells were washed three times, 100 μl of TMB substrate was added and incubated 15 min at room temperature. Subsequently, 50 μl of stop solution was added and the absorbance value (expressed as optical density [OD]) was read by an ELISA plate reader. The result of tests were defined as follows: OD values > 0.1, positive; OD values ≤0.1, negative, the tests would be repeated by using 100 μl serum samples.

### Antibody production

For the preparation of anti-HSV-1 monoclonal antibodies, six-week-old female BALB/c mice were immunized with KOS viruses by standard prime and boost vaccination procedure. The hybridoma cells were obtained following the protocol reported previously [40]. The positive clones were screened and selected by indirect ELISA coated with KOS viruses and immunostaining assay that the antibodies can specifically bind to the HSV-1 infected cells. MAbs were prepared by ascites production and purified by using protein G chromatography according to the manufacturer’s instructions.

### ELISPOT

U-2 OS cells were seeded in 96-well plates at a density of 2 × 10^4^ cells per well and incubated for 6 h at 37 °C in 5 % CO_2_ until the cells became monolayer. 1 × 10^4^ PFU viruses in a volume of 100 μL were added to the monolayer cells and incubated for 14 h. After infection, the cells were fixed with 1 % paraformaldehyde in PBS for 5 min and followed by permeabilization with 0.1 % Triton X-100 in PBS at room temperature for 5 min. Next, each diluted monoclonal antibody (10 μg/mL) was added and incubated at 37 °C for 1 h. After reaction with the HRP-conjugated secondary antibody (1:2000), the spots were developed in TMB substrates (Sigma) within 15 min. Last, the plates were spin dried and counted using ImmunSpot@S5 UV Analyzer (Cellular Technology Limited). Each antibody was repeated in triplicate wells and each test was carried out in duplicate or triplicate. An irrelevant antibody, which specifically recognized HBV envelope protein, was used as a negative control.

### Western Blotting

Western blotting was done as previously described [41]. In brief, cells were lysed in lysis buffer (20 mM Hepes, 1 mM EGTA, 5 mM MgCl2, 100 mM NaCl, 4 mg/mL Decyl-beta-d-maltopyranoside, 10 % glycerol and protease inhibitor cocktail). Cell lysates were separated by SDS-PAGE and transferred onto a nitrocellulose membrane. After membranes were blocked with 5 % BSA for 1 h, they were probed with indicated primary antibodies (8 μg/mL) overnight at 4 °C, followed by incubation with the HRP-conjugated secondary antibodies for 1 h at room temperature, and finally visualized with the Lumi-Light^PLUS^ Western blotting Substrate (Roche).

### Immunofluorescence

Standard immunofluorescence staining was performed as previously described [40]. In brief, cells on the glass slides were fixed with 1 % paraformaldehyde and permeabilized by 0.1 % Triton X-100. After blocked with 5 % normal goat serum in PBS for 1 h, the slides were stained with primary antibody 2G5 (8 μg/mL) overnight at 4 °C, then incubated with FITC conjugated secondary antibody before examination using confocal microscope.

### Plaque reduction neutralization test

U-2 OS cells were seeded in 6 cm dishes at a density of 1 × 10^6^ cells per well and cultured for 2 days at 37 °C in 5 % CO_2_ until the cells became monolayer. All serum samples were heat inactivated at 56 °C for 30 min prior to testing. The test serum samples were first diluted 20-fold in DMEM containing 10 % heat inactivated FBS, followed by two-fold serial dilutions from 1:40 to 1:10240. An equal volume of viral dilution, containing 100 PFU viruses, was mixed with the diluted serum and incubated at 37 °C in 5 % CO_2_ for 1 h. Then the total mixture in a volume of 0.5 mL per dish was added evenly to the U-2 OS cells and incubated for 1.25 h. During the incubation, the infected dishes were shaken gently every 15 min. After the initial infection, the inoculums were removed and 4 mL fresh culture medium were added to facilitate the viral entry. After another 2 h incubation, the cell monolayers were overlaid with 10 mL of 2 % methylcellulose medium and then incubated at 37 °C in 5 % CO_2_ for 3 days. Last, the dishes were stained with 5 mL of 0.01 % neutral red for overnight, then the plaques were counted by use of a white-light transilluminator manually. Virus control wells were infected with the same amount of virus as the testing wells mixed with serially diluted serum. Each serum dilution was repeated in triplicate wells and each test was carried out in duplicate or triplicate. The raw data of each sample by PRNT method was calculated in a formula as follows: % reduction = 100 × [1-(average plaque counts for each dilution/average plaque counts for virus control) [16]. If the % reduction were below 50 %, the serum samples were retested by starting a dilution from 5-fold.

### ELISPOT based neutralization test

U-2 OS cells were seeded in 96-well plates at a density of 2 × 10^4^ cells per well and incubated for 6 h at 37 °C in 5 % CO_2_ until the cells became monolayer. Serial dilutions of serum samples were prepared as described for PRNT with minor modifications. 1 × 10^4^ PFU viruses in a volume of 50 μL were mixed with an equal volume of serial diluted serum samples and incubated at 37 °C in 5 % CO_2_ for 1 h in 96-well plates with U bottom shape. Then the mixture was added to the monolayer cells and incubated for 14 h. The optimal infectious dose and incubation time were determined in preliminary experiments in order to acquire reliable spot counts. Then, standard ELISPOT procedure was performed as previously described. Each serum dilution was repeated in triplicate wells and each test was carried out in duplicate or triplicate. The reduction rate of the serum sample can be calculated in the same manner as for PRNT.

### Data analysis

The 50 % inhibitory concentration (IC_50_) of each sample is defined as the highest dilution that gave 50 % plaque reduction. The neutralizing antibody titers can be interpreted as the highest serum dilution that neutralizes 50 % of the viruses (NT_50_), which was calculated by nonlinear, dose–response regression analysis using Graphpad Prism software. The correlation between the two methods was analyzed by linear regression using SPSS 16.0 software. The average NAb titers across different ages were compared using two-tailed Mann–Whitney *U* test. A *P* value of less than 0.05 was considered statistically significant, and the confidence interval (CI) was 95 %.

### Ethics

The study was conducted in accordance with the ethical principles of the Declaration of Helsinki and was approved by the Human Ethics Committee of the Xiamen University. Informed consent was obtained from each patient. The use of mice was approved by the Institutional Animal Care and Use Committee at Xiamen University.
